# Phytochemical Compositions and Biological Activities of Essential Oils from the Leaves, Rhizomes and Whole Plant of *Hornstedtia bella* Škorničk

**DOI:** 10.3390/antibiotics9060334

**Published:** 2020-06-18

**Authors:** Matthew Gavino Donadu, Nhan Trong Le, Duc Viet Ho, Tuan Quoc Doan, Anh Tuan Le, Ain Raal, Marianna Usai, Mauro Marchetti, Giuseppina Sanna, Silvia Madeddu, Paola Rappelli, Nicia Diaz, Paola Molicotti, Antonio Carta, Sandra Piras, Donatella Usai, Hoai Thi Nguyen, Piero Cappuccinelli, Stefania Zanetti

**Affiliations:** 1 Department of Biomedical Sciences, University of Sassari, 07100 Sassari, Italy; mdonadu@uniss.it (M.G.D.); rappelli@uniss.it (P.R.); ndiaz@uniss.it (N.D.); molicott@uniss.it (P.M.); pcappuc@gmail.com (P.C.); zanettis@uniss.it (S.Z.); 2 Department of Chemistry and Pharmacy, University of Sassari, 07100 Sassari, Italy; dsfusai@uniss.it (M.U.); acarta@uniss.it (A.C.); piras@uniss.it (S.P.); 3 Faculty of Pharmacy, Hue University of Medicine and Pharmacy, Hue University, Hue 49000, Vietnam; ltrongnhan@hueuni.edu.vn (N.T.L.); hvietduc@hueuni.edu.vn (D.V.H.); doanquoctuan@hueuni.edu.vn (T.Q.D.); 4 Mientrung Institute for Scientific Research, VAST, Hue 49000, Vietnam; tasa207@gmail.com; 5 Institute of Pharmacy, Faculty of Medicine, University of Tartu, 50900 Tartu, Estonia; ain.raal@ut.ee; 6 Institute of Biomolecular Chemistry (CNR), Li Punti, 07100 Sassari, Italy; mauro.marchetti@ss.icb.cnr.it; 7 Department of Biomedical Sciences, University of Cagliari, 09042 Monserrato, Italy; g.sanna@unica.it (G.S.); silvia.madeddu@unica.it (S.M.)

**Keywords:** essential oils, infections, antifungal activity, antimicrobial activity

## Abstract

The rapid emergence of drug-resistant strains and novel viruses have motivated the search for new anti-infectious agents. In this study, the chemical compositions and cytotoxicity, as well as the antibacterial, antifungal, antitrichomonas, and antiviral activities of essential oils from the leaves, rhizomes, and whole plant of *Hornstedtia bella* were investigated. The GC/MS analysis showed that β-pinene, *E*-β-caryophyllene, and α-humulene were found at high concentrations in the essential oils. The essential oils exhibited (i) inhibition against *Staphylococcus aureus,* methicillin-resistant *Staphylococcus aureus*, *Staphylococcus epidermidis* with minimum inhibitory concentrations (MIC) and minimum lethal concentration (MLC) values from 1 to 4% (*v/v*); (ii) MIC and MLC values from 2 to 16% (*v/v*) in *Candida tropicalis* and *Candida parapsilosis*; (iii) MIC and MLC values from 4 to 16% in *Enterococcus faecalis*; and (iv) MIC and MLC values from 8 to greater than or equal to 16% (*v/v*) in the remaining strains, including *Escherichia coli*, *Pseudomonas aeruginosa*, *Klebsiella pneumoniae*, *Candida albicans,* and *Candida glabrata*. In antitrichomonas activity, the leaves and whole-plant oils of *Hornstedtia bella* possessed IC_50_, IC_90_, and MLC values of 0.008%, 0.016%, and 0.03% (*v/v*), respectively, whilst those of rhizomes oil had in turn, 0.004%, 0.008%, and 0.016% (*v/v*).Besides, the leaf oil showed a weak cytotoxicity against Vero 76 and MRC-5; meanwhile, rhizomes and whole-plant oils did not exert any toxic effects on cell monolayers. Finally, these oils were not active against EV-A71.

## 1. Introduction

Infectious diseases are caused by infectious microorganisms, including bacteria, viruses, fungi, and parasites [1]. A broad range of antibiotics have been produced to inhibit or kill microorganisms, thus playing a key role in the treatment of many infectious diseases [2]. However, the rapid emergence of drug-resistant strains has hindered the effectiveness of this therapy [3,4]. Viruses have been mutating and transforming into new species, many of which have caused devastating consequences, namely, acquired immunodeficiency syndrome by HIV (AIDS), severe acute respiratory syndrome (SARS) by coronavirus, hemorrhagic fevers by Ebola virus [1], and most recently, the pandemic crisis of COVID-19 by SARS-CoV-2, which has affected millions of people worldwide [5]. For enterovirus A71 (EV-A71), outbreaks of EV-A71 have been frequently reported since 1969, but a series of EV-A71 epidemics in the Asia–Pacific region (Australia, Japan, Malaysia, Taiwan, Vietnam and China) between 1997 and 2010 have raised a particular concern about the potential emergence of EV-A71 as a worldwide health threat. Recently, EV-A71 epidemics also occurred in European countries, such as the Netherlands, France, and Spain [6]. Despite many efforts to stop the spreading, the numbers of antibiotics for curing these infectious diseases are still limited and the process of manufacturing new antibiotic usually takes a very long time to be commercially ready [1]. Also, the utility of synthetic chemicals to control microorganisms is challenging owing to their toxicity, environmental hazards, and carcinogenic characteristics [7]. Therefore, it is very crucial to search for an effective alternative replacing the use of synthetic compounds whilst exerting promising effects on the treatment of infectious diseases [8]. In this regard, plant-derived substituents have emerged as exceptional anti-infectious agents providing a range of potent biological benefits and safe use. One of the most prevalent plant-based products are essential oils (EOs) as a result of their remarkable biological and pharmacological effects, thus having a profound impact on the therapeutic treatments of a range of diseases [8].

Eos (ethereal oils or volatiles) are aromatic oily liquids extracted from various parts of plants such as flowers, roots, leaves, seeds, buds, wood and fruits [9], via many methods, including expression, fermentation, enfleurage or extraction and steam distillation, of which steam distillation has been most widely adopted [9]. Angiospermic families include several species, such as Zingiberaceae, Rutaceae, Asteraceae, Lamiaceae, and Myrtaceae which are usually rich sources of EOs. EOs are referred to as secondary metabolites of aromatic plants, also known as by-products of plant metabolism [10]. More than 3000 EOs have been reported, nearly 300 of which provide many promising effects on a range of aspects, including pharmacy, food industry, cosmetics, and perfumes [8]. EOs were found to exert various biological activities, such as antimicrobial, antioxidant, anti-inflammatory, and anticancer properties [8].

The Zingiberaceae family has been known as the largest family under Zingiberales, comprising 50 genera and more than 1600 species. These species are distributed mainly in tropical and subtropical Asia, Africa, Australia, and the Americas [11]. Members of Zingiberaceae are flower plants, perennial herbs with creeping horizontal or tuberous rhizomes, which usually grow in moist shady regions [11,12]. The Zingiberaceae species are well-known for their medicinal and economic significances [13]. Many species are used for indigenous medicinal herbs, spices, food, flavoring agents, cosmetics, and decoration [14]. From the species of this family, a myriad of volatile oils and oleoresins are obtained, thus enabling a great deal of medical contributions across India and Asian countries. For example, EOs from *Zingiber officinale*, *Curcuma longa*, *Alpinia galangal* and *Curcuma zanthorrhiza* have long been utilized in traditional medicine in many centuries [15]. *Zingiber officinale* oil has been used in medicine and perfumery [13]. *Alpinia officinarum* roots, rhizomes of *Alpinia galanga*, *Curcuma longa* and *Zingiber officinale*, and seeds of *Elettaria cardamomum* and *Amomum cardamomum* have been known as spices, condiments and flavoring agents [13]. Especially, curcumin extracted from *Curcuma longa* has been recognized and used worldwide for multiple potential health benefits [16].

The extracts of Zingiberaceae rhizomes contain many EOs consisting of numerous complex compound mixtures, such as terpenoid, alcohols, ketones, phytoestrogens, and flavonoids [17]. There have been many previous studies identifying the chemical compositions and biological activities of EOs from Zingiberaceae species [18,19,20,21,22,23].

*Hornstedtia* Retzius, Observ., is a genus of plants in the Zingiberaceae composed of 40 known species [24]. Several species of the genus *Hornstedtia* have been reported for chemical compositions and biological activities of EOs [25,26,27,28]. Among the two species of the genus *Hornstedtia* found in Vietnam, we attempted to investigate *Hornstedtia bella* Škorničk (*H. bella*). *H. bella* is a large terrestrial rhizomatous herb, distributed in the forests of central Vietnam. It is usually grown near streams or in other humid locations, on slate or granite. Flowers of *H. bella* are white with light pink tones at the top, flowering from April to June [29]. Inflorescences and sometimes also young leafy shoots, which are strongly aromatic, are used for the preparation of soups and various dishes (indigenous knowledge). The word “bella” was chosen to name this species because it is one of the most beautiful Vietnamese species [29]. Until now, there has not been any research on the chemical compositions and biological activity of compounds extracted from *H. bella*. Thus, to provide information about *H. bella,* we carried out the current study to identify the chemical compositions and biological activities of EOs from *H. bella*.

The present work aimed to study the chemical compositions of EOs from leaves, rhizomes and the whole plant of *H. bella* collected in Thua Thien Hue Province, Vietnam, and assess their cytotoxicity and potential antiviral, antibacterial, antimycotic and antitrichomonas activities. To the best of our knowledge, this is the first scientific report about the chemical compositions and pharmacological properties of EOs from *H. bella*. 

## 2. Results

### 2.1. Extraction Yield and Chemical Compositions of Essential Oils

The extract of EOs appeared as a pale-yellow oily liquid. The average yields of EOs were calculated based on dry weight, affording 0.35 ± 0.01%, 0.24 ± 0.01% and 0.27 ± 0.01% (v/w) for leaves, rhizomes and whole plant, respectively. The GC/MS analysis showed that the whole plant oil contained 26 constituents representing 96.2% of the total oil content (Table 1). The main classes of compounds in this oil were monoterpene hydrocarbons (36.98%), sesquiterpene hydrocarbons (31.80%), oxygenated sesquiterpenes (15.95%), and oxygenated monoterpenes (11.47%). The constituents accounted for higher amounts in the oil from the whole plant of *H. bella* were β-pinene (25.52%), 1,8-cineole (10.50%), α-pinene (8.75%), *E*-β-caryophyllene (5.56%), α-humulene (5.64%), germacrene D (5.84%) and ledene (5.11%). For leaves oil, the main classes of compounds included sesquiterpene hydrocarbons (69.72%), oxygenated sesquiterpenes (18.82%), and monoterpene hydrocarbons (7.27%). The main constituents in the leaves oil of *H. Bella* were germacrene D (17.47%), viridiflorene (11.13%), *E*-β-caryophyllene (7.32%) and α-humulene (6.48%). In the rhizomes oil were identified 56 compounds (95.25% of total). The main classes of compounds found in the rhizomes oil were sesquiterpene hydrocarbons (32.55%), monoterpene hydrocarbons (28.79%) and oxygenated sesquiterpenes (24.21%). The main constituents found in rhizomes were totally different with respect to such found in leaves. In fact, β-pinene (16.07%), α-humulene (9.68%), β-selinene (7.11%) and epiglobulol (6.48%) were the main constituents in the rhizomes oil. The only diterpene found in leaves was retinal (2.14%). 

### 2.2. Antimicrobial Activities

The antimicrobial activities of EOs from leaves, rhizomes, and whole plant of *H. bella* are displayed in Table 2. In general, the rhizomes oil exhibited better antibacterial and antifungal activities than the leaves oil. On the other hand, three EOs showed higher sensitivities to Gram-positive than Gram-negative bacteria. For *Candida* species, EOs from leaves and rhizomes of *H. bella* indicated the best antifungal activity against *Candida tropicalis* (*C. tropicalis*) and *Candida parapsilosis* (*C. parapsilosis*). In fact, the leaves oil of *H. bella* displayed: (i) strongest inhibition against *Staphylococcus aureus* (*S. aureus*)*,* methicillin-resistant *S. aureus* (MRSA), *Staphylococcus epidermidis* (*S. epidermidis*) and *C. tropicalis* with MIC of 2% (*v/v*) and MLC of 4%(*v/v*); (ii) efficiency against *C. parapsilosis* with MIC and MLC values of 4% (*v/v*); (iii) MIC and MLC values from 8 to greater than or equal to 16% (*v/v*) in the remaining strains. Meanwhile, the rhizomes oil of *H. bella* illustrated: (i) highest inhibition against *S. aureus*, MRSA and *S. epidermidis* with MIC of 1% (*v/v*) and MLC of 2% (*v/v*); (ii) sensitivity against *C. tropicalis* and *C. parapsilosis* with MIC and MLC values were both 2% (*v/v*); (iii) MIC of 4% and MLC of 8% (*v/v*) in *Enterococcus faecalis* (*E*. *faecalis*); (iv) MIC and MLC values from 8% to more than or equal to 16% (*v/v*) in the remaining strains. In addition, the whole plant’s oil shown (i) strongest inhibition against *S. aureus*, MRSA and *S. epidermidis* with MIC and MLC values from 2 to 4% (*v/v*); (ii) MIC and MLC values from 8% to more than or equal to 16% (*v/v*) in the remaining strains. 

The ratio of MLC/MIC has been widely adopted to evaluate the microbicidal or microbiostatic action, with, for example, a value less than or equal to 4.0 exhibiting microbicidal effects and a value above 4.0 indicating microbiostatic action [3]. Herein, the MLC/MIC ratios obtained from the experimental data were all less than 4, proving that both leaf and rhizome EOs of *H. bella* possessed bactericidal and fungicidal properties on the studied strains. 

### 2.3. Antitrichomonas Activity

Table 3 illustrates that EOs from leaves, rhizomes, and whole plants of *H. bella* induced cytotoxicity to *Trichomonas vaginalis* (*T. vaginalis*), with IC_50_, IC_90,_ and MLC values that were highly dependent on the incubation time. Here, EOs exhibited a relatively weak effect after 1 h of incubation but then rapidly increased over time. Particularly, rhizome oil possessed a more profound impact on *T. vaginalis* than leaf oils. Following the introduction of the leaves, rhizomes and whole-plant EOs at 24 h, anti-trichomonas activity was observed to increase by 64-, 16-, and 32-times, respectively, compared to those at 1 h; which in turn, continued to increase by 2-, 4-, and 2-times at 48 h compared to 24 h. At this time point, the leaves and whole plant EOs of *H. bella* also had IC_50_, IC_90_, and MLC values at 0.008%, 0.016% and 0.03% (*v/v*), respectively, whilst those of rhizomes oil were 0.004%, 0.008%, and 0.016% (*v/v*). 

### 2.4. Cytotoxicity and Antiviral Activity

The potential antiviral activity of *H. bella* EOs was tested in cell-based assays against an important human pathogen, Enterovirus A71, while their cytotoxicity was evaluated in parallel assays using uninfected cell lines. As shown in (Table 4), unfortunately, EOs were not endowed with anti-EVA71 activity nor cytotoxicity against normal human Lung fibroblasts (MRC-5) and monkey kidney cells (Vero 76). However, leaf oil showed weak cytotoxic against the selected cell monolayers with CC_50_ of 80 and 100 µg/mL, respectively.

## 3. Discussion

EOs are composed of 20–60 compounds and the major components can constitute nearly 85% of an EO [38,39]. The chemical compositions of EOs are greatly influenced by the place of origin, climatic conditions, plant species and seasons [40,41]. EOs extracted from plants are stored in their non-differentiated cells or secreted organs, including cavities, secretory ducts and glandular hairs. Sometimes, several components of EOs are not readily present in the plants itself but are rather generated from the hydrolysis of several compounds found in the plants [42]. Besides, some constituents of the EOs were products of decomposing processes of the previous components during distillation [42]. In this study, three EOs showed large amounts of β-pinene, *E*-β-caryophyllene, and α-humulene. Germacrene D and ledene were found at high concentrations in the oil of leaves but very little that of rhizomes. Meanwhile, β-selinene, epiglobulol, and eudesm-7(11)-en-4-ol were found in high quantities in the oil of rhizomes and almost none of these were present in leaf oil. The cause for these differences may be due to the variation of enzyme systems in leaves and rhizomes, as well as the impact of environmental factors above and below the ground that affect the formation of EO components [42]. In addition, some components were found at high concentrations in rhizome oil but not in whole-plant oil, such as β-selinene, epiglobulol, and eudesm-7(11)-en-4-ol. Since these two EOs were extracted from samples that were collected at two different times, seasonal variation in EOs compositions are thought to be the main cause of this difference [43,44]. Furthermore, other parts of the whole plant, including flowers and stems, can provide a great source of these compounds. Moreover, the comparison of chemical compositions of EOs from *H. bella* and *Hornstedtia sanhan* [25] which is another unique species belonging to *Hornstedtia* genus in Vietnam, showed a similarity in the chemical compositions of their EOs, particularly α-pinene, β-pinene, β-caryophyllene and α-humulene, which were found as their major components. The main constituents of *H. bella* EOs, such as β-pinene, α-humulene, germacrene D, and β-selinene were also observed to be abundantly present in the EOs from *Hornstedtia havilandii* and *Hornstedtia scyphifera* [26,27]. Despite the presence of some of these volatile molecules in EOs of other genera, EOs extracted from species in the same genus still share the same predominant chemical substituents, indicating that they have an important chemotaxonomic relevance [8].

The roles of EOs in plants are diverse, for example, in pollination or defense mechanisms, often as a repellent or irritant. It has been found that EOs can be great sources of antioxidants donating hydrogen in oxidative reactions, especially in the presence of light. EOs were also documented to exhibit antifungal and antibacterial properties, thus protecting the plant from possible pathogenic dangers [45]. Each component in EOs represents different mechanisms of action on microorganisms [9]. These mechanisms synergize with one another, ultimately resulting in many effective antimicrobial properties of EOs [46]. In fact, EOs with different chemical compositions tend to break down bacteria and fungi in different pathways [7]. EOs can inhibit bacteria in various approaches, including triggering the degradation of bacterial cell walls, causing leakage of cell contents, damage to membrane proteins, disruption of cytoplasmic membranes, depletion of the proton motive force or the coagulation of cytoplasms [9]. -Also, the hydrophobicity of EOs facilitates the increasing permeation of EOs into cell membranes, thus leveraging the spillage of ions and molecules out of the cells and causing cell apoptosis [8]. 

Of all the bacteria investigated, the leaf and rhizome EOs from *H. bella* displayed enhanced inhibitory activities against Gram-positive compared to Gram-negative bacteria. Gram-negative bacteria indeed have more complex cell walls than Gram-positive bacteria, thereby, the approach of EOs towards Gram-positive bacteria was effectively preferential [8]. However, the rhizome oil of *H. bella* exhibited better antibacterial and antifungal activities than those of leaf oil. This can be explained by the difference in chemical compositions of these EOs [3], especially the main components, as they play an important role in determining the ultimate biological properties of the EOs [47]. The antibacterial activity of EOs from *H. bella* was likely due to its major compounds such as germacrene D, viridiflorene, E-β-caryophyllene, and α-humulene. E-β-caryophyllene has shown antimicrobial activity against bacteria and fungi [48]. α-Humulene has shown antibacterial activity against *S. aureus* [49]. According to Ricardo et al. [50], large amounts of (E)-caryophyllene and germacrene D were found in *Verbenaceae* species, in which *Verbenaceae virgata* oil exhibited antimicrobial activity against *S. aureus* and *E. coli*. EOs from *Duguetia gardneriana* and *Duguetia moricandiana* containing high levels of germacrene D, viridiflorene, and β-caryophyllene have been shown to act against *S. aureus* [51]. 

In comparison with previous studies in the antimicrobial activities of the EOs from *Hornstedtia* species, Siti et al. indicated that *Hornstedtia havilandii*-extracted EOs can act against *S. aureus* with an MIC of 112.5 µg/mL, whilst having weak effects on *E. coli* and *P. aeruginosa*, *C. albicans* and *C. glabrata* [26]. The leaf, rhizome, and flower EOs from *Hornstedtia scyphifera* exhibited moderate activities against Gram-positive bacteria with MIC values of 225–450 µg/mL [27]. 

*S. aureus* is one of the main pathogens of nosocomial infections, and many other infective diseases, namely endocarditis, bacteremia, osteoarticular and pleuropulmonary. *S. aureus* involves the infection of the skin and soft tissue, and causes device-related infections, of which bacteremia is the most prevalent [52]. However, the emergence of MRSA strains has been hampering the efficacy of current antibiotics. On the other hand, EOs, thanks to their exciting biological efficiencies and greatly natural safety, have been widely adopted to obstruct both methicillin-sensitive *S. aureus* (MSSA) and MRSA strains [53]. Indeed, Halcon et al. [54] indicated that *Melaleuca alternifolia* oil was effective against *S. aureus* and MRSA. Recently, we showed that EOs from *Leoheo domatiophorus* and *Paramignya trimera* inhibited *S. aureus* with MIC values of 0.25 and 2% (*v/v*), respectively [3,8]. The standard and clinical isolates of MRSA and MSSA were inhibited by *Zataria multiflora* oil at a range of concentrations from 0.55 to 1.41 µL/mL [53]. In the current study, EOs from leaves, rhizomes, and whole plants of *H. bella* displayed inhibition against *S. aureus* and MRSA with MIC and MLC values from 1 to 4% (*v/v*). 

*Candida* is a relatively common yeast in the human mucosa, including the digestive tract, reproductive tract, and oral cavity. About 80% of the healthy population may be susceptible to a fungal infection like *Candida*. *Candida* infections often have a broad spectrum, ranging from a superficial oral thrush and vaginitis to candidemia, which can be severely dangerous [55]. Fluconazole is a triazole antifungal, most largely used to treat *Candida* infections. Fluconazole is fungistatic rather than fungicidal and thus likely to create an ideal environment for its resistant strains to emerge [56]. In fact, some clinical isolates of *Candida* species have been developing great resistance against fluconazole as well as other conventional usees of triazole antifungal drugs; hence, the demand for newly effective antifungals is utterly required [53]. In the concept of using naturally rooted compounds with biological effects, EOs have long been known as potent fungal inhibitors [57]. Some EOs showed antimicrobial activity against *Candida* species, such as tea tree [58,59], clove, thyme [57], cinnamon, lemongrass, Japanese mint, geranium, motiarosha and ginger grass EOs [60]. Moreover, an important property of EOs is the protection of plants from pathogens but the resistance of microorganisms to EOs has not been observed [57]. *C. parapsilosis* and *C. tropicalis* were more sensitive to the leaves and rhizomes EOs of *H. bella* compared to *C. albicans* and *C. glabrata*, whilst whole-plant oil from *H. bella* did not show any significant antimicrobial activity against the tested *Candida* species in our previous studies [61,62] and the experimental results obtained in this case. The sensitivity may vary depending on the chemical compositions of EOs and the species of *Candida*. The current work indicated that EOs from leaves and rhizomes of *H. bella* were effective against *C. parapsilosis* and *C. tropicalis* with MIC and MLC values from 2 to 4% (*v/v*). 

Enterovirus 71 (EV-A71) is a human pathogen responsible for several diseases ranging from mild infections like herpangina to severe neurological disorders. Epidemics of various scales are recurrent in the Asia–Pacific region [63]. With the aim to assess the potential anti EVA71 activity of our *H. bella*-originated EOs, we performed an in vitro assay. However, these oils were not active against EV-A71. In parallel, we evaluated cytotoxicity against normal human cells and monkey cells that support viral replication (Vero 76). Leaf oil from *H. bella* showed weak cytotoxicity against Vero 76 and MRC-5 in the high µg/mL range (CC_50_ = 80 and 100 µg/mL). On the contrary, rhizomes and whole plant EOs did not exert any toxic effects on cell monolayers with CC_50_ values over 100 µg/mL (Table 4).

*T. vaginalis* is the cause of one of the most widely non-viral sexually transmitted infections around the world [64]. The World Health Organization (WHO) estimated 156 million cases of *T. vaginalis* infection worldwide in 2016, accounting for almost half of sexually transmitted infection incidence in the globe [65]. *T. vaginalis* can cause urethral and prostate infections in men. About 50% of women infected with *T. vaginalis* have no obvious symptoms, but it can cause some serious consequences, such as infertility, premature birth, premature rupture of membranes, and death in infants [64]. In addition, trichomoniasis increases the risk of human immunodeficiency virus (HIV) infection [66]. For decades, metronidazole has been the first choice to treat *T. vaginalis* infections [67]; however, the emergence of drug-resistant strains is limiting the effectiveness of this therapy [68,69]. Therefore, a promising alternative of using plant-based products for the treatment of *T. vaginalis* and other parasites has appeared to be of great benefit to improving the therapeutic proficiency [70]. From 1981 to 2010, more than half of anti-parasitic drugs were nature-based products [71]. Mozhgan et al. reported that 95 in vitro and clinical studies have investigated the anti-trichomonas activity of compounds and extracts from plants [72]. There have been several studies describing some EOs against *T. vaginalis*, such as EOs from *Paramignya trimera*, *Limnocitrus littoralis* [3], *Leoheo domatiophorus* [8], *Marrubium vulgare* [70], *Ocimum basilicum* [73], *Lavandula angustifolia*, *Lavandula intermedia* [74] and *Amomum tsao-ko* [75]. In the current study, the leaf, rhizome, and whole-plant EOs of *H. bella* were found to be effective against *T. vaginalis* with MLC values of 0.03, 0.016 and 0.03% (*v/v*), respectively, after 48 h of incubation. Noticeably, the effect of rhizome EOs against *T. vaginalis* was rapidly observed only 1 h following incubation represented by the values of IC_50_, IC_90_ and MLC at 0.25, 0.5 and 1% (*v/v*), respectively. In contrast, 5 h treatment with metronidazole was needed for the DNA synthesis in *T. vaginalis* to be inhibited and then cell death [66]. Moreover, metronidazole enters *T. vaginalis* cells by passive diffusion and the slow drug metabolism reduces the difference of drug concentration in and out the cells; hence, reducing the absorption of metronidazole into cells [66]. However, the penetration of metronidazole can be favored when combined with EOs of *H. bella*, probably due to the great hydrophobicity of EOs that facilitates the absorption of metronidazole; this combination may also provide a synergistic anti-infectious effect; thus amplifying the ultimate treatment efficiency. 

In summary, in the present work, we identified the chemical compositions and investigated cytotoxicity, potential antiviral, antibacterial, antimycotic and anti-trichomonas activities of EOs from *H. bella*. The choice of microorganisms under study was carried out taking into account their related pathologies and emphasizing the importance of finding new sources of active compounds that can inhibit or slow down their proliferation. However, we have not studied the mechanism of action of *H. bella* EOs. Instead, the current research provides an overview of the strains of microorganisms inhibited by *H. bella* EOs. Hence, there remain a range of experiments that can be implemented in future studies, such as testing on other cells or pathogens, investigating pharmacological effects of the main components of *H. bella* EOs, elucidating the mechanisms of action of EOs from *H. bella* and their major chemical constituents, studies on resistant strains, assessing the toxicity and safety of *H. bella* EOs, and an in vivo investigation to confirm the encouraging results of this work. 

## 4. Materials and Methods

### 4.1. Plant Material

Plant samples of *H. bella* were collected in Luoi District, Thua Thien Hue Province, Vietnam (16°18′04.3″ N 107°13′07.0″ E). The whole plant was collected for the first time in April 2019, whilst leaves and rhizomes were collected a second time in September 2019. Dr. Chinh Tien Vu, Vietnam National Museum of Nature, identified the plant. Three voucher specimens (LHB-01, RHB-02 and THB-03) were deposited at Hue University of Medicine and Pharmacy, Vietnam. 

### 4.2. Extraction of the Essential Oils

EOs were hydrodistilled for 3.5 h at ambient pressure using a Clevenger-type apparatus [76] from leaves, rhizomes and whole plant of shredded *H. bella*. The extraction yields (three replications) were calculated on dry materials. The EOs were dried on Na_2_SO_4_ and stored at 4 °C before using in chemical analysis and biological assays. 

### 4.3. Analysis of the Essential Oils

Samples were analyzed by a *Hewlett–Packard Model 5890A* gas chromatography (GC) fitted with a 60 m × 0.25 mm, thickness 0.25 μm *HP-5* fused SiO_2_ capillary column. Injector and detector temperatures at 280 °C [8]. 

GC oven temperature was programmed as follows: from 50 to 135 °C at 5 °C/min (1 min), 5 °C/min to 225 °C (5 min), 5 °C/min to 260 °C, held for 10 min [8]. 

The EOs were analyzed without dilution (using 2,6-dimethylphenol as an internal standard) and injected by a split/splitless automatic injector. The percentage of each compound was referred to absolute weight using internal standard and response factors [8].

Mass spectrometry (MS) analyses were carried out using an *Agilent Technologies* model 7820A associated with an MS detector 5977E MSD (Agilent), at the same column and analytical conditions used for GC analyses. The HP-5 column was linked to the ion source of the mass spectrometer. Mass units were monitored from 10 to 900 at 70 eV [8]. 

The retention indices (RI) of single compounds were determined by co-injection with a homologous series of *n*-alkanes (C_9_–C_22_) [77].

### 4.4. Antimicrobial Activities

In the present work, we selected 17 microbial strains, including 3 reference strains and 14 clinical isolates as: (i) Gram-positive strains (*Staphylococcus aureus*, methicillin-resistant *Staphylococcus aureus*, *Staphylococcus epidermidis* and *Enterococcus faecalis*); (ii) Gram-negative strains (*Escherichia coli, Pseudomonas aeruginosa* and *Klebsiella pneumonia)*; *Candida* species strains (*Candida albicans*, *Candida glabrata*, *Candida tropicalis* and *Candida parapsilosis*). Cultures were maintained in appropriate media at 4 °C. The cells were cultivated at 37 °C on agar plates for 18 h prior to experiments.

### 4.5. Determination of Minimum Inhibitory Concentrations (MIC) and Minimum Lethal Concentration (MLC)

In order to establish the MIC and MLC of bacteria and *Candida* species, the broth dilution method was employed as reported by the Clinical and Laboratory Standard Institute [78]. The inoculum was prepared by diluting colonies in salt solution at a concentration of 0.5 McFarland, then recorded at *λ* 530 nm by a spectrophotometric reading. The EOs solutions were diluted to different concentrations from 16% to 0.06% (*v/v*). After shaking, oil dilution (100 μL, each) and bacterial/yeast suspension (100 μL at 10^6^ CFU/mL) were added to each well and then incubated within 24, 48 h at 37 °C for bacteria, fungi, respectively. MIC values were evaluated by the lowest concentration of the EOs at which bacterial growth is visibly inhibited after overnight incubation. In order to determine the MLC values, 10 μL were seeded on Mueller Hinton agar and Sabouraud Dextrose agar and the plates were incubated within 24 h for bacteria and 48 h for fungi at 37 °C. MLC value is the lowest concentration that reduces the viability of the initial microbial inoculum by ≥99.9%. Each assay was undertaken with a positive growth control consisting of organisms in broth, and a negative sterility control containing uninoculated broth. Each experiment was performed in duplicate and repeated three times.

### 4.6. Antitrichomonas Activity 

*T. vaginalis* strain G3 was cultured axenically in vitro by daily passages in Diamond’s Trypticase Yeast extract Maltose (TYM) medium (Sigma Chemical Co., St. Louis, MO, USA) plus 10% fetal bovine serum (FBS) at 37 °C in a 5% CO_2_ atmosphere [79]. Exponentially growing *T. vaginalis* cells were harvested and viability was assessed by microscopy. Trichomonas cells (viability >95%) were centrifuged at 1500 rpm for 10 min and resuspended in Diamond’s TYM medium at 2 × 10^5^ cells/mL [80].

EOs from leaves, rhizomes and whole plant of *H. bella* were serially diluted in 100 μL of Diamond’s TYM medium from 16% to 0.002% (*v/v*) in 96-well plates. The prepared trichomonad suspension (100 μL) was then added to each well. Diamond’s TYM medium alone was used as a growth control. The culture plate was kept at 37 °C in a CO_2_ incubator and checked after 1, 4, 24, and 48 h. The percentage of viable *T. vaginalis* cells was observed by microscope. The MLC was defined as the lowest EOs concentration at which no viable protozoa were observed. The 50% inhibitory concentration (IC_50_) and ≥90% inhibitory concentration (IC_90_) values were considered as the EOs concentration at which 50% and ≥90% of *T. vaginalis* cells were killed, respectively. Each assay has been repeated independently at least two times [75].

### 4.7. Cells and Cytotoxicity Assays

MRC-5 cells, human lung fibroblasts from normal tissue [ATCC CCL-171], and Vero 76, Monkey kidney [ATCC CRL 1587] were purchased from American Type Culture Collection (ATCC, Manassas, Virginia, USA). Cell cultures were checked periodically for the absence of mycoplasma contamination with MycoTect Kit (Gibco).

Human lung fibroblasts were seeded at 1x10^6^ cells/mL in 96 well plates in Minimum Essential Medium with Earle’s salts (MEM-E) medium with L-glutamine, supplemented with 10% FBS, 0.025g/L kanamycin. Vero-76 cells were seeded at an initial density of 4 × 10^5^ cells/mL in 96-well plates, in culture medium Dulbecco’s Modified Eagle Medium (D-MEM) with L-glutamine, supplemented with FBS, 0.025g/L kanamycin. Cells were then incubated at 37 °C in a humidified, 5% CO_2_ atmosphere in the absence or presence of serial dilutions of *H. bella* EOs. Cell viability was determined after 72 (MRC-5) and 96 (Vero 76) hrs at 37 °C by the 3-(4,5- dimethylthiazol-2-yl)-2,5-diphenyl-tetrazolium bromide (MTT) method [81]. Dimethyl sulfoxide (DMSO) was used as a control in each experiment, and it was tested at the maximum concentration present in each compound. The cytotoxicity of test compounds (100 μg/mL, the maximum concentration tested) was evaluated in parallel with their antiviral activity.

### 4.8. Viruses and Antiviral Assay

Single-stranded RNA (ssRNA+) Enterovirus A71 strain BrCr [ATCC VR-1775] was purchased from American Type Culture Collection (ATCC). EVA71 was maintained and propagated in appropriate cell lines and stored in small aliquots at −80 °C until use.

Essential oil’s activity against EVA71, was determined by plaque reduction assays in infected cell monolayers as described previously [82]. Briefly, the monolayer of Vero-76 cells was grown overnight on a 24-well plate. The cells were then infected for 2 h with proper virus dilutions to give 50–100 PFU/well. After removal of unadsorbed virus, cells were overlayed with 500 μL of medium [D-MEM with L-glutamine and 4500 mg/L D-glucose, supplemented with 1% inactivated FBS] containing 0.75% methyl-cellulose and serial dilutions of test products. Medium was also added to not treat wells as non-infection controls. Cultures were incubated at 37°C for 4 days and then fixed with PBS containing 50% ethanol and 0.8% crystal violet, washed and air-dried. The number of plaques in the control (no inhibitor) and experimental wells were then counted.

### 4.9. Statistical Analysis and Linear Regression Analysis 

Data processing was performed in analysis of variance (ANOVA) using software MSTAT-C, and mean separation was conducted using the least significant difference (LSD) test at *p* ≤ 0.05 level of significance.

The degree of cell growth/viability and viral multiplication at each given drug concentration, were presented as percentage of untreated controls. Concentrations inducing 50% inhibition (CC_50_ or EC_50_) were identified by linear regression analysis. 

## 5. Conclusions

In conclusion, the chemical compositions and biological activities of EOs from leaves, rhizomes and whole plant of *H. bella* were investigated. β-Pinene, *E*-β-caryophyllene and α-humulene were found at high concentrations in three EOs. The EOs displayed strongest inhibition effects against *S. aureus*, MRSA, *S.epidermidis*, *C. tropicalis* and *C. parapsilosis*. The EOs also demonstrated inhibition against *T. vaginalis*. The leaf oil showed a weak cytotoxicity against Vero-76 and MRC-5 cells, while rhizome and whole-plant EOs did not exert any toxic effects on cells monolayers. These EOs were not active against EV-A71. Further studies should be done on resistant strains, assessing the toxicity and safety of *H. bella* EOs in vivo. 

## Figures and Tables

**Table 1:** Chemical compositions of the EOs from *H. bella.*

No.	RT	^a^ KI	Components	^b^ % ± SD			^c^ IM	^d^ Ref.	^e^ CID
				Leaves	Rhizomes	Whole Plant			
1	18.21	939	α-Pinene	1.43 ± 0.09	4.96 ± 0.11	8.75 ± 0.15	Std		6654
2	19.15	954	Camphene		4.28 ± 0.09	1.22 ± 0.05	Std		6616
3	20.71	979	β-Pinene	4.61 ± 0.10	16.07 ± 0.37	25.52 ± 0.43	Std		14896
4	23.43	1029	Limonene	0.17 ± 0.02	1.45 ± 0.11	1.49 ± 0.09	Std		22311
5	23.63	1031	1,8-Cineole	0.90 ± 0.07	2.03 ± 0.10	10.50 ± 0.11	Std		2758
6	29.82	1149	Camphor	-	1.56 ± 0.07	-	Std		2537
7	30.22	1150	Camphene hydrate	-	0.29 ± 0.03	-	MS-RI	[30]	101680
8	31.05	1169	Borneol	-	0.97 ± 0.06	-	Std		64685
9	31.44	1177	Terpinen-4-ol	-	0.31 ± 0.02	-	Std		11230
10	32.14	1189	α-Terpineol	0.11 ± 0.01	1.46 ± 0.13	0.97 ± 0.09	MS-RI	[31]	17100
11	36.43	1289	Bornyl acetate	-	0.88 ± 0.05	-	Std		6448
12	38.45	1347	1,5,5-Trimethyl-6-methylene-cyclohexene	0.16 ± 0.01	-	-	MS		578237
13	39.01	1351	α-Cubebene	0.08 ± 0.01	-	-	Std		84609
14	39.9	1375	α-Ylangene	0.12 ± 0.02	-	-	MS		442409
15	40.13	1377	α-Copaene	0.53 ± 0.10	0.43 ± 0.09	-	Std		442355
16	40.46	1388	β-Bourbonene	0.39 ± 0.07	-	-	MS		62566
17	40.55	1391	β-Elemene	0.27 ± 0.04	0.32 ± 0.08	-	MS		6918391
18	41.28	1410	α-Gurjunene	0.08 ± 0.01	2.39 ± 0.017	1.47 ± 0.14	MS		15560276
19	41.77	1419	*E*-β-Caryophyllene	7.32 ± 0.35	4.08 ± 0.08	5.56 ± 0.23	Std		5281515
20	42.02	1432	β-Copaene	0.29 ± 0.01	-	-	Std		57339298
21	42.34	1441	Aromadendrene	0.42 ± 0.03	-	-	MS		91354
22	42.49	1441	*cis*-α-Ambrinol	0.34 ± 0.02	-	-	MS		24858722
23	42.55	1448	10s,11s-Himachala-3(12),4-diene	0.37 ± 0.02	-	-	MS		14038471
24	42.64	1450	*cis*-Muurola-3,5-diene	0.55 ± 0.09	0.31 ± 0.04	-	MS		51351708
25	42.74	1454	*trans*-Muurola-3,5-diene	1.06 ± 0.07	-	-	MS		102512379
26	42.91	1455	α-Humulene	6.48 ± 0.19	9.68 ± 0.09	5.64 ± 0.21	Std		5281520
27	43.05	1460	Alloaromadendrene	2.26 ± 0.04	0.29 ± 0.02	1.08 ± 0.04	MS		91354
28	43.44	1480	γ-Muurolene	3.79 ± 0.06	1.42 ± 0.04	1.50 ± 0.04	MS-RI	[32]	12313020
29	43.45	1483	α-Elemene		0.43 ± 0.01	-	MS		10583
30	43.59	1485	α-Amorphene	1.33 ± 0.04	-	1.09 ± 0.05	MS		101708
31	43.75	1485	Germacrene D	17.47 ± 0.17	1.08 ± 0.03	5.84 ± 0.013	MS		5317570
32	43.83	1490	β-Selinene		7.11 ± 0.09	-	MS		442393
33	43.84	1493	δ-Selinene	0.78 ± 0.04	-	0.36 ± 0.02	MS		10123
34	43.99	1496	Valencene		0.37 ± 0.01	-	MS		9855795
35	44.02	1496	Ledene (viridiflorene)	11.13 ± 0.08	-	5.11 ± 0.10	MS-RI	[33]	10910653
36	44.07	1498	α-Selinene		2.20 ± 0.14	-	Std		10856614
37	44.08	1500	α-Muurolene	2.89 ± 0.07	-	0.74 ± 0.04	Std		12306047
38	44.26	1502	Epizonarene	0.78 ± 0.04	-	-	MS-RI	[34]	595385
39	44.34	1512	δ-Amorphene	1.92 ± 0.12	-	-	MS		10223
40	44.39	1512	*cis*-γ-Cadinene	0.84 ± 0.05	1.07 ± 0.02	-	MS		6429304
41	44.42	1514	γ-Cadinene	1.73 ± 0.07	-	-	MS		6432404
42	44.55	1522	7-epi-α-Selinene	1.48 ± 0.13	-	0.54 ± 0.04	MS		10726905
43	44.66	1523	δ-Cadinene	3.84 ± 0.11	1.07 ± 0.9	2.87 ± 0.11	MS		441005
44	44.77	1529	*trans*-Calamenene	0.41 ± 0.03	0.30 ± 0.02	-	MS		6429022
45	44.82	1530	Zonarene	0.86 ± 0.09	-	-	MS-RI	[34]	6428488
46	45.13	1532	Epiglobulol	0.09 ± 0.01	6.48 ± 0.09	-	MS		11858788
47	45.22	1550	*cis*-Muurola-5-en-4-β-ol	0.27 ± 0.01	-	-	MS		91749819
48	45.36	1566	β-Calacorene	0.14 ± 0.02	-	-	MS		529621
49	45.94	1568	Cadala-1(10),3,8,triene	0.11 ± 0.02	-	-	MS		593889
50	46.27	1571	Palustrol	0.47 ± 0.04	-	-	MS-RI	[35]	110745
51	46.45	1578	Spathulenol	2.55 ± 0.08	1.21 ± 0.9	3.22 ± 0.17	Std		92231
52	46.62	1583	Caryophyllene oxyde	-	0.88 ± 0.03	-	MS		14350
53	46.71	1585	Globulol	2.40 ± 0.10	0.71 ± 0.04	2.48 ± 0.09	MS		12304985
54	46.95	1593	Viridiflorol (ledol)	1.12 ± 0.08	0.46 ± 0.06	1.30 ± 0.09	MS		11996452
55	47.22	1596	β-Eudesmol	1.28 ± 0.07	0.40 ± 0.03	1.06 ± 0.04	MS		91457
56	47.35	1608	α-Humulene epoxide II	0.17 ± 0.01	2.10 ± 0.04	-	MS		5363694
57	47.71	1616	Epicubenol	1.31 ± 0.04	0.78 ± 0.02	1.27 ± 0.07	MS-RI	[36]	12046149
58	47.86	1620	Isospathulenol	1.01 ± 0.02	-	1.07 ± 0.02	MS-RI	[37]	14038848
59	47.92	1625	Guaiol	-	0.57 ± 0.01	-	Std		227829
60	48.04	1640	α-epi-Cadinol	2.10 ± 0.08	0.31 ± 0.02	-	Std		12302222
61	48.10	1640	α-epi-Muurolol	1.10 ± 0.04	2.17 ± 0.12	1.31 ± 0.09	Std		3084331
62	48.15	1642	Cubenol	0.58 ± 0.02	-	-	Std		519857
63	48.25	1642	γ-Cadinol	-	1.42 ± 0.06	-	Std		91753503
64	48.41	1654	α-Cadinol	3.53 ± 0.03	1.25 ± 0.04	4.24 ± 0.05	Std		10398656
65	48.47	1654	β-Cadinol	-	0.43 ± 0.01	-	Std		12302231
66	48.52	1682	Ledene-oxide-(II)	0.84 ± 0.02	1.21 ± 0.04	-	MS		534497
67	48.55	1684	Eudesm-7(11)-en-4-ol	-	3.83 ± 0.08	-	MS		6432454
68	50.62	1927	8-(2-Acetyloxiran-2-yl)-6,6-dimethylocta-3,4-dien-2-one	-	1.59 ± 0.04	-	MS		539293
69	52.07	1939	2-Methyl-4(2,6,6-trimethyl ciclohen-1-enyl)but-2-en-1-ol	-	0.50 ± 0.02	-	MS		569166
70	60.49	2184	*Z*-Retinal	-	2.14 ± 0.05	-	MS		6436082
			**Total**	**96.26**	**95.25**	**96.2**			
			**Monoterpene hydrocarbons**	**7.27**	**28.79**	**36.98**			
			**Oxygenated monoterpenes**	**0.11**	**5.47**	**11.47**			
			**Sesquiterpene hydrocarbons**	**69.72**	**32.55**	**31.80**			
			**Oxygenated sesquiterpenes**	**18.82**	**24.21**	**15.95**			
			**Diterpenes**	**0**	**2.14**	-			
			**Others**	**0.34**	**2.09**	-			

Data represent mean values ± SD for three independent determinations. ^a^ Retention indices relative to *n*-alkanes series. ^b^ Content of components. **^c^** Identification methods (IM): **MS** by comparison of the mass spectrum with those of the available mass libraries Adams, NIST 11, and by interpretation of the fragmentations in mass spectra. **RI** by comparison of retention index with those reported in literature. **Std** by comparison of the retention time and mass spectrum of available authentic standards. ^d^ Papers provided data in order to compare the relative RI. ^e^ PubChem: https://pubchem.ncbi.nlm.nih.gov.

**Table 2:** Antimicrobial activities (MIC and MLC) of EOs from *H. bella.*

Strains	Leaves Oil		Rhizomes Oil		Whole Plant Oil	
	MIC (% *v/v*)	MLC (% *v/v*)	MIC (% *v/v*)	MLC (% *v/v*)	MIC (% *v/v*)	MLC (% *v/v*)
**Gram-Positive Bacteria**						
*Staphylococcus aureus* ATCC 43300	2 ± 0.5	4 ± 0.5	1 ± 1	2 ± 1	4 ± 0.5	4 ± 0.5
Methicillin-resistant *S. aureus* clinical	2 ± 1	4 ± 0.5	1 ± 1	2 ± 1	2 ± 0.5	4 ± 0.5
Methicillin-resistant *S. aureus* clinical	2 ± 1	4 ± 0.5	1 ± 1	2 ± 1	2 ± 0.5	4 ± 0.5
Methicillin-resistant *S. aureus* clinical	2 ± 1	4 ± 0.5	1 ± 1	2 ± 1	2 ± 0.5	2 ± 0.5
Methicillin-resistant *S. aureus* clinical	2 ± 1	4 ± 0.5	1 ± 1	2 ± 1	2 ± 0.5	2 ± 0.5
*Staphylococcus epidermidis* clinical	2 ± 1	4 ± 0.5	1 ± 1	2 ± 1	2 ± 0.5	4 ± 0.5
*Staphylococcus epidermidis* clinical	2 ± 1	4 ± 0.5	1 ± 1	2 ± 1	2 ± 0.5	4 ± 0.5
*Enterococcus faecalis* clinical	8 ± 0.5	16 ± 0.5	4 ± 1	8 ± 1	8 ± 0.5	8 ± 0.5
**Gram-Negative Bacteria**						
*Escherichia coli* ATCC 35218	>16 ± 0.5	>16 ± 0.5	16 ± 0.5	>16 ± 0.5	>16 ± 0.5	>16 ± 0.5
*Escherichia coli* clinical	>16 ± 0.5	>16 ± 0.5	16 ± 0.5	16 ± 0.5	>16 ± 0.5	>16 ± 0.5
*Pseudomonas aeruginosa* ATCC 27853	>16 ± 0.5	>16 ± 0.5	16 ± 0.5	16 ± 0.5	16 ± 0.5	>16 ± 0.5
*Pseudomonas aeruginosa* clinical	>16 ± 0.5	>16 ± 0.5	>16 ± 0.5	>16 ± 0.5	16 ± 0.5	>16 ± 0.5
*Klebsiella pneumoniae* clinical	>16 ± 0.5	>16 ± 0.5	16 ± 0.5	16 ± 0.5	16 ± 0.5	>16 ± 0.5
**Yeast**						
*Candida albicans* 556 RM	>16 ± 0.5	>16 ± 0.5	8 ± 0.5	8 ± 0.5	16 ± 0.5	>16 ± 0.5
*Candida glabrata* clinical	16 ± 0.5	16 ± 0.5	8 ± 1	8 ± 1	16 ± 0.5	16 ± 0.5
*Candida tropicalis* 1011 RM	2 ± 1	4 ± 1	2 ± 1	2 ± 1	16 ± 0.5	16 ± 0.5
*Candida parapsilosis* RM	4 ± 0.5	4 ± 0.5	2 ± 0.5	2 ± 0.5	16 ± 0.5	16 ± 0.5

MIC and MLC values represent the mean ± SD of three independent experiments.

**Table 3:** In vitro anti-*T. vaginalis* activity of EOs from *H. bella.*

Time	Leaf Oil			Rhizome Oil			Whole-Plant Oil		
	IC_50_	IC_90_	MLC	IC_50_	IC_90_	MLC	IC_50_	IC_90_	MLC
**1 h**	1	2	4	0.25	0.5	1	0.5	1	2
**4 h**	0.12	0.25	0.5	0.12	0.25	0.5	0.12	0.25	0.5
**24 h**	0.016	0.03	0.06	0.016	0.03	0.06	0.016	0.03	0.06
**48 h**	0.008	0.016	0.03	0.004	0.008	0.016	0.008	0.016	0.03

Data represent mean values for two independent experiments. IC_50_ (% *v/v*): The concentration that causes 50% *T. vaginalis* growth inhibition. IC_90_ (% *v/v*): The concentration that causes ≥ 90% *T. vaginalis* growth inhibition. MLC (% *v/v*)**:** The concentration that causes the death of 100% *T. vaginalis*.

**Table 4:** Cytotoxicity and antiviral activity of EOs from leaves and rhizomes of *H. bella* against selected cell lines and Enterovirus A71 (EVA71).

Cell Lines and Virus	MRC-5 ^a^	Vero 76 ^b^	EVA71
	CC_50_^c^		EC_50_^d^
Leaves oil	100	80	>80
Rhizomes oil	>100	>100	>100
Whole-plant oil	>100	>100	>100
Rupintrivir ^e^			0.07

Data represent mean values ± SD for three independent determinations. For values where SD is not shown, variation among triplicate samples was less than 15%. ^a^ Normal human lung fibroblast; ^b^ Monkey kidney; ^c^ Compound concentration (µg/mL) required to reduce cell proliferation by 50%, as determined by the MTT method; ^d^ Compound concentration (µg/mL) required to reduce the plaque number of EV-A71 by 50% in Vero-76 monolayers; ^e^ Reference compound: EC_50_ is in µM.
